# Oral Semaglutide Therapy Reduces Cardiovascular Events in Patients with Type 2 Diabetes: Deciphering the Soul of the Study

**DOI:** 10.3390/jcm14103335

**Published:** 2025-05-11

**Authors:** Ishwarlal Jialal, Samuel T. Olatunbosun

**Affiliations:** 1 Departments of Internal Medicine and Pathology, UC Davis School of Medicine, Davis, CA 95618, USA; 2 Endocrinology Section, Medical Service, VA Northern California Health Care System, Mather, CA 95655, USA; 3 Division of Endocrinology, Diabetes and Metabolism, Department of Internal Medicine, University of California Davis Health, Sacramento, CA 95817, USA

Glucagon-like peptide-1 receptor agonists (GLP-IRAs) have become pivotal in the management of type 2 diabetes mellitus (T2DM).Their actions include stimulating insulin secretion and inhibiting glucagon secretion from the endocrine pancreas, decreasing appetite and increasing satiety in the brain, decreasing gut motility and delaying gastric emptying in the gastro-intestinal tract, and decreasing hepatic glucose production [1,2,3,4]. The majority of GLP-1RAs are injectable which limits adherence and greater usage.

Oral semaglutide (Rybelsus) was ushered in as a novel delivery system for peptides and approved for the management of T2DM.This novel formulation combines semaglutide with an absorption enhancer, sodium N-(8-[2-hydroxybenzoyl]amino) caprylate (SNAC). SNAC provided a gastric pH that protects the peptide from acid hydrolysis and enhances gastric absorption by increasing transcellular permeability of the gastric epithelium by temporarily opening tight junctions [5]. Importantly, oral semaglutide has to be taken once daily in the fasting state with 120 mL of water and the patient has to wait for 30 min before consuming food or taking other medications. Also, a dose-escalation strategy is required to reduce gastro-intestinal side effects [5].

Oral semaglutide is used as an adjunct or an alternative monotherapy for patients with type 2 diabetes in whom initial therapy with lifestyle intervention and metformin has failed or those unable to take metformin. The R1 formulation (3, 7, and 14 mg tablets) was initially approved by the U.S. Food and Drug Administration (FDA) in September 2019, and subsequently, in January 2023, permission for its use as a first-line medication was granted.

In December 2024, the FDA approved the R2 formulation (1.5, 4, and 9 mg tablets) of oral semaglutide as an adjunctive agent to improve glycemic control in T2DM. The new formulation was developed with enhanced absorption, allowing for lower doses to achieve comparable efficacy with the R1 formulation. The use of lower initial dose(s) of each formulation was aimed at reducing GI symptoms, as they do not provide effective glycemic control.

Oral semaglutide is utilized particularly in patients with T2DM with or at risk of atherosclerotic cardiovascular disease (ASCVD), when weight loss is desired, and/or in patients with glycated hemoglobin (HbA1c) relatively far from goal levels. Some clinicians see an early role for oral semaglutide in newly diagnosed drug-naive T2DM based on studies that support the initiation of combination therapy to achieve glycemic targets more quickly compared with a stepwise approach to pharmacotherapy.

In the Peptide Innovation for Early Diabetes Treatment (PIONEER) 6 trial, oral semaglutide was demonstrated to be non-inferior to placebo in T2DM patients with high cardiovascular risk, suggesting cardiovascular safety. The sample size was 3183, with 1591 patients receiving semaglutide with a very short mean duration of 15.9 months [6].

Recently, the results of the large Semaglutide Cardiovascular Outcomes Trial (SOUL) were published [7]. This was a double-blind, placebo-controlled superiority trial conducted in 33 countries across 5 continents. The patients were ≥50 years of age with T2DM (median duration of 14.7 years) with a glycated hemoglobin (HbA1c) concentration between 6.5 and 10%. Also, the patients had to have one of the following conditions: coronary artery disease, cerebrovascular disease, symptomatic peripheral arterial disease, or chronic kidney disease (CKD), defined as a glomerular filtration rate of <60 mL/min/1.73 m^2^. Patients received oral semaglutide, the R1 formulation (maximum dose 14 mg/d), or placebo. The primary outcome was a composite of major atherosclerotic cardiovascular (ASCVD) events including cardiovascular death, nonfatal myocardial infarction, and nonfatal stroke. The confirmatory secondary end point included a 5-point composite of major kidney disease events [7]. Patients with end-stage CKD and/or who had received long-term kidney replacement therapy were excluded. In addition to standard care, patients received semaglutide in an escalating dose to minimize side effects (3 mg, 7 mg, and 14 mg) or matching placebo, with a 14 mg dose being maintained until the end of the trial.

The mean age of the participants was 66.1 years, and 71.5% were male (28.5% female). White people comprised 69% of the patients, and the mean body mass index was 31 kg/m^2^ with a mean HbA1c concentration of 8%. Also, median LDL cholesterol was 73.5 mg/dL, triglycerides 159 mg/dL, and hsCRP 2.0 mg/L. The median follow-up was 49.5 months with an interquartile range of 44–55 months.

The primary end point, which was a 3-point composite including cardiovascular death, nonfatal myocardial infarction, and nonfatal stroke, occurred with a 12% incidence in the oral semaglutide group and 13.8% in the placebo group: hazard ratio (HR) = 0.86 with 95% confidence intervals (CIs) of 0.77–0.96, *p* = 0.006. This finding demonstrates the superiority of oral semaglutide compared to placebo.

Non-fatal myocardial infarction occurred less frequently in the oral semaglutide group, occurring in 4% versus 5.2%, respectively: HR = 0.74, CI ranging from 0.61 to 0.89. There were no significant differences in nonfatal stroke between the two groups, occurring in 3 vs. 3.3% (HR = 0.88, CI of 0.70–1.11), or in cardiovascular mortality between the two groups, occurring in 6.2% vs. 6.6% (HR = 0.93 with CI of 0.80–1.09).

There was no significant benefit in the occurrence of major kidney disease events (including the 5-point composite), with 8.4% incidence vs. 9.0%: HR = 0.91, CI of 0.8–1.05, *p* = 0.19. Also, there was no improvement in heart failure or total mortality.

There were significant decreases in HbA1c at 104 weeks vs. baseline, with a −0.71% decrease in the semaglutide group. Also, there were significant differences compared to baseline in body weight of −4.2 kg and hsCRP level of 1.56 mgL vs. 2.0 mg/L. Importantly, there were no increases in the number of episodes of severe hypoglycemia in the oral semaglutide group.

Gastrointestinal (GI) side effects were more common with oral semaglutide and were the major reason for discontinuation. Infections were more common in the placebo group. Supplementary Table 4 [7] details the adverse events. Neoplasms (both benign and malignant) were significantly increased with oral semaglutide: 6.8% vs. 5.7%. Also, there was a significant increase in gall bladder disease: 2.8% vs 2.2%.

Observation of a very high frequency of GI adverse effects (AEs) with the R1 formulation was noted in the SOUL study, with an incidence of 6.4% versus 2.0% in oral semaglutide and placebo, respectively, and more importantly, clinical experience necessitated the development of the R2 formulation, which was not tested in this trial. Whether this development will make oral semaglutide more acceptable and improve adherence to therapy remains to be seen. For instance, the rates of GI AEs between oral semaglutide and once-weekly injectable semaglutide with a dose of 1 mg were shown to be similar in a randomized clinical trial, but real-life experience seems to suggest otherwise.

There has been a notion that the glycemic and weight benefits achievable with oral semaglutide treatment would overcome the negative perception of the side effects. The reality is that the dose-proportionality of the gastrointestinal AEs of oral semaglutide potentially limits the percent of patients that can reach the optimal dosage, constituting a barrier to maximum drug effectiveness [8].

The available clinical data on the risk of developing malignant tumors while patients are on injectable semaglutide have largely been reassuring. The increased rate of neoplasms noted in the oral semaglutide group in the SOUL study deserves further exploration.

What can we conclude about oral semaglutide therapy?

In conclusion, oral semaglutide clearly has a role in the management of T2DM, although when compared with the injectable, it seems that there has been some trade off with clinical efficacy; also, the higher occurrence of GI AEs may limit its use in clinical practice.

In this large trial, oral semaglutide therapy resulted in a 14% relative risk reduction in major ASCVD events compared to placebo in T2DM patents with ASCVD, CKD, or both. This appears to be largely driven by a reduction in nonfatal myocardial infarction. These findings cannot be readily translated to the usual T2DM patient seen in our clinics. Furthermore, only 28.5% of the participants in the study were female, and there was a gross under-representation of Black patients, making up only 2.6%, despite South Africa and the US being two of the countries included. Also, there was no significant improvement in the occurrence of major CKD events. This contrasts with injectable semaglutide and could be due to lower bioavailability [9].

Surprisingly, the authors make no comment on plausible mechanisms to explain the reduction in ASCVD events, although other investigators have hypothesized that reductions in weight, HbA1c, CRP, systolic blood pressure, and LDL cholesterol levels could be potential mediating mechanisms [8,10]. Finally, cost, especially in the developing countries, will be a major issue.

## Data Availability

The data are available from the senior author for review on reasonable request.
